# Experimental Study of Cement Alkali-Resistant Glass Fiber (C-ARGF) Grouting Material

**DOI:** 10.3390/ma13030605

**Published:** 2020-01-29

**Authors:** Zhenyue Shi, Qingbiao Wang, Lei Xu

**Affiliations:** 1 College of Mining and Safety Engineering, Shandong University of Science and Technology, Qingdao 266590, China; shihongyue@126.com; 2 State Key Laboratory of Mining Disaster Prevention and Control Co-Founded by Shandong Province and the Ministry of Science and Technology, Shandong University of Science and Technology, Qingdao 266590, China; 3 National Engineering Laboratory for Coalmine Backfilling Mining, Shandong University of Science and Technology, Tai’an 271019, China; 4 Department of Resources and Civil Engineering, Shandong University of Science and Technology, Tai’an 271019, China; 5 Tiezheng Testing Technology Co., Ltd., Jinan 250014, China; xulei4232010@163.com

**Keywords:** Alkali-resistant glass fibers, grouting material, compressive strength, tensile strength, flexural strength, impervious performance

## Abstract

Mixing alkali-resistant glass fiber (ARGF) into grouting slurry can prevent the development of cracks; thus, understanding the properties of ARGF grouting material is important for applications in engineering. Two types of ARGFs (Cem-FIL®60 and Anti-Crak®HD) were selected as mixing materials, and their performance was tested in four areas, namely, compressive strength, tensile strength, flexural strength, and impervious performance, under four different mixing amounts of fiber (0%, 0.25%, 0.5%, and 1.0%). Results demonstrate that the addition of ARGF increased the compressive strength and tensile strength of the grouting slurry, and the best performance was at 0.5%. The effect on the flexural strength and impervious performance was related to the mixing amount, and the fiber may have induced a counter-effect for certain amounts of added ARGF. Mixing ARGF could increase the early strength ratio of grout; however, a high early strength ratio did not necessarily result in high strength, as the flexural strength did not change synchronously with the early strength ratio; a similar pattern was found for the impermeability. Cem-FIL®60 had a better effect on the properties of grouting materials than Anti-Crak®HD. These results were successfully applied in the water-plugging and reinforcement engineering of a karst tunnel.

## 1. Introduction

Grouting is one of the most effective methods used to solve geological problems, and the characteristics of a grouting material can have a direct and critical impact on the effectiveness of grouting. While the development of geological structures is complex, the diversity in the environment contributes to the richness and diversity of grouting materials. Different grouting materials have different advantages with different applications. Therefore, mastering the properties of new grouting materials is the key to ensuring the effectiveness of grouting. In order to improve the effectiveness of grouting materials, many researchers conducted studies on the development and performance of grouting materials with rich and distinctive results.

Research and development of chemical grouting materials. He et al. [1] studied the influence of a silane coupling agent, 3-chloropropyl trimethoxysilane, on the mechanical properties of polyurethane/water glass grouting materials through mechanical tests such as compression tests and fracture mechanics tests. Faramarzi et al. [2] analyzed the effect of using urea-formaldehyde resin as an additive in the grouting process to improve slurry parameters. Duan et al. [3] developed a cement-modified urea-formaldehyde resin double slurry grouting material, adding an alkaline coagulant. The test showed that the strength of the stone decreased with an increase in the coagulant. Pan et al. [4] studied the failure mode and grouting effect of silica sol grout. Wang et al. [5] studied the effect of the aluminum sulfate and quicklime/fluorogypsum ratio on the grouting material and determined the optimal mix ratio of the grouting material.

Development of special geological grouting materials. Kim et al. [6] developed materials and grouting methods suitable for grouting in saturated parabolic layers. Sha et al. [7] developed a new type of high-efficiency ultra-fine cement-based grouting material to solve the grouting problem of water-rich sand layers and experimentally tested the new grouting material containing high strength and other advantages. The SH-(C + F + CaO) grouting fluid developed by Chen et al. [8] had a good effect on the fracture grouting of soil sites in northwestern China. Zhang et al. [9] studied the grouting material of calcined ginger nuts and earthen site soil, and analyzed the relationships among the flexural strength, compressive strength, permeability coefficient, and density of the grouting material in detail. Zhang et al. [10,11] developed an environmentally friendly controllable paste grouting material with short setting time, rapid yield stress growth, high fluidity, and high early strength, which was applied in the karst tunnel crossing the river of Changsha metro line 3. They also studied the stability of filling grouting slurry.

Research on environmentally friendly grouting materials. Park et al. [12] studied the substitution of biological grouting for cement to reduce cement consumption and protect the environment. Ortega et al. [13,14] studied the microstructure and durability of the grouting materials of fly ash and slag cement mortar and the long-term effect of a sulfate environment on fly ash and slag cement. Nowoświat et al. [15] studied the effect of fly ash amount on the rheological properties of fresh mortar. Wei et al. [16] studied the effect of water content on the mechanical strength and microstructure of alkali-activated fly ash and ground granulated blast furnace slag mortar.

Perez-Garcia et al. [17] mixed green cementing mortar with grouting materials to provide a grouting solution in which slag is used instead of cement. Lee et al. [18] developed cementless grouting materials grouting material and studied its properties and mix ratio, laying a theoretical foundation for the replacement of ordinary Portland cement material grouting. Liu et al. [19] carried out a grouting reinforcement test for karst-filled clay, and the results showed that grouting not only increased the initial cracking strength of the soil but also improved the cohesion and permeability of the soil.

Research on fiber grouting materials. Nishimura et al. [20] studied the effect of nanofibers on enhancing the mechanical properties of cement and improve the flexural, compressive, and tensile strength of glass ionomer cement. Wang et al. [21] studied the flow pattern of glass fiber cement slurry, determined the flow pattern of the glass fiber under different amounts of glass fiber, and successfully applied it under the conditions of karst flowing water. This study could also provide theoretical guidance for the reinforcement of grouting in coal mines under deep and complex geological conditions [22,23,24,25,26]. Zhu et al. [27,28] established a damage constitutive model for alkali-resistant glass fiber (ARGF)-reinforced concrete, and they made reasonable predictions regarding its damage. Liu et al. [29] studied water-soluble polyurethane grouting material modified by hydroxypropyl methyl cellulose. This material has good resistance to erosion and is suitable for grouting under dynamic water conditions. Park [30] studied the tensile strength characteristics of fiber-reinforced cement paste in rock and soil. The test results showed that, for an increase in the fiber content from 0% to 1%, its tensile strength increased by 119–190% and 23–131% at 7 d and 28 d, respectively.

Although much research on grouting materials and their properties was conducted, the main areas of focus were on chemical grouting materials, special geological grouting materials, and some environmentally friendly grouting materials. However, there was very little research on fiber grouting materials, whereas the existing research mainly focused on nano fiber and polyurethane fiber as added material in terms of their effect on the properties of slurry injection. Few studies were done on the flow pattern of alkali-resistant glass fiber grout, whereas the compressive strength, tensile strength, flexural strength, and impermeability of alkali-resistant glass fiber grouting materials were not studied. ARGF is an environmentally friendly material with excellent alkali resistance and corrosion resistance. The addition of ARGF can avoid the adverse effect of cement alkaline corrosion on the admixture, improving the performance of grouting materials to a certain extent. ARGF can play a role in preventing crack and impermeability, as well as improve the grouting effect. Therefore, it is necessary to carry out experimental research on the properties of ARGF grouting materials in order to grasp the characteristics of the influence of the type and amount of materials, as well as the effect of other conditions on the grouting materials.

In this study, laboratory tests and engineering application methods were used. Two ARGFs, Cem-FIL60 and HD, were selected to study the compressive strength (CS), tensile strength (TS), flexural strength (FS), and impervious performance (IP) of ARGF with four mixing amounts (0%, 0.25%, 0.5%, and 1.0%). The performance of the slurry with mixed ARGF in terms of CS, TS, FS, and IP, as well as its early strength ratio of CS (ECSR), early strength ratio of TS (ETSR), early strength ratio of FS (EFSR), and early permeant ratio (EPR), was analyzed. In addition, the nature of the influence of material types and mixing amount on grouting performance was obtained. The material was applied to karst tunnel grouting projects, and it achieved good grouting results, providing an important reference for the application of ARGF grouting slurry.

## 2. Performance Test of Cement Alkali-Resistant Glass Fiber (C-ARGF) Grout Material

### 2.1. Test Materials and Equipment

**Figure 1:**
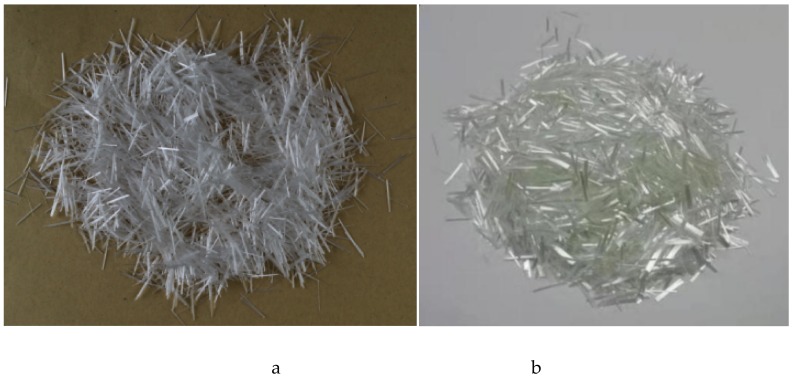
Two specimens of alkali-resistant glass fiber (ARGF): (**a**) Cem-FIL60 ARGF; (**b**) HD ARGF.

**Figure 2:**
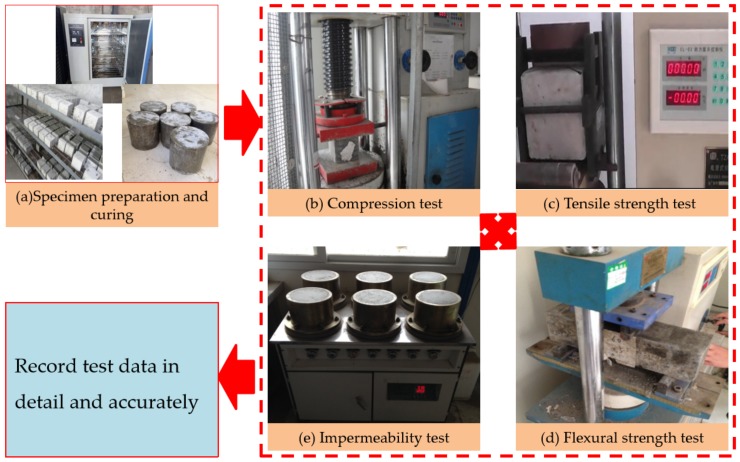
Test arrangement and procedure.

The cement used was PC 32.5R composite Portland cement produced by Sinoma Zhuzhou Cement Co., Ltd., while alkali-resistant chopped strands produced by Taishan Glass Fiber Co., Ltd. (Tai’an, China) were used for the ARGF. Cem-FIL60 and HD were selected, and their characteristic parameters are shown in Table 1, while Figure 1 shows the ARGF specimen. The main equipment selected for the laboratory test is listed in Table 2, and Figure 2 shows a physical view of the device.

### 2.2. Test Scheme

#### 2.2.1. Slurry Mix Ratio and Experimental Group Design

##### Mix Ratio Design

C-ARGF was made by mixing water, cement, and ARGF in a certain proportion. To make the study of the strength properties of slurry stones representative, the water–cement ratio used in this experiment design was 1:1 (water–cement ratio refers to the ratio of the weight of water to that of cement). When the water–cement ratio is 1:1, C-ARGF grout is characterized as a Bingham fluid [21,31]. The high water–cement ratio and ARGF make C-ARGF slurry suitable for grouting in karst areas by avoiding its dilution by karst water to some extent. This is conducive to ensuring grouting quality. Referring to technical specifications [32], the commonly used volume proportion of synthetic fiber is 0.06–0.3%, and the maximum volume proportion should not exceed 1.5%. Thus, the mixing amounts of Cem-FIL60 and HD glass fiber materials were set to 0%, 0.25%, 0.5%, and 1.0%, whereby the ARGF mixing amount was the volume proportion.

##### Design of Experimental Group

In order to facilitate specimen classification, specimens adulterated with Cem-FIL-type of glass fiber grout are represented by CFIL (e.g., CFIL-0.25 refers to a Cem-ARGF content of 0.25%). Specimens adulterated with HD-type ARGF are represented by HD, and the slurry specimens without ARGF are classified as NCH-0. For CS and TS testing, the specimen size was 100 mm (length) × 100 mm (width) × 100 mm (height). For the FS test, the specimen size was 400 mm (length) × 100 mm (width) × 100 mm (height). For the IP test, the specimens were circular-truncated cones, with a size of 175 mm (top diameter) × 185 mm (basal diameter) × 150 mm (height). According to the specification requirements [33,34], the specific experimental group design is shown in Table 3.

#### 2.2.2. Test Method

The slurry configuration, specimen preparation, and hardening were carried out at a room temperature of 20 ± 5 °C. When the specimen hardening time reached 24 h, the mold was removed; then, the specimens were put into a 20 ± 2 °C environment for curing. The test was conducted after the specimens reached the test time, according to the maintenance requirements. At present, there are many testing methods for glass fiber cement products in the world [35,36]. This test adopted the Chinese CECS13:2009 standard and its auxiliary standards [37,38,39,40]. This test for CS, TS, and FS is similar to the testing method commonly used; thus, it is not described further. 

In terms of the impermeability test, the seepage height method was adopted. The Vaseline-coated specimens were pressed into a test mold, which was installed on the HP-4.0 impermeability tester, as shown in Figure 2e. Then, a pressurized water test was conducted under 0.5 MPa water pressure. After testing for 24 h, no water emerged from any of the specimens. The specific test flow is shown in Figure 2.

## 3. Results and Discussions

By adopting the above experimental methods, the experimental results of CS, TS, FS, and PS for each experimental group are obtained, the results are displayed using intuitive graphics, and a systematic analysis is performed. The values of CS, TS, and FS are the average values of the three specimens in each group, and the value of impermeability height is the average height of the six specimens in each group.

### 3.1. Experimental Results of CS

**Figure 3:**
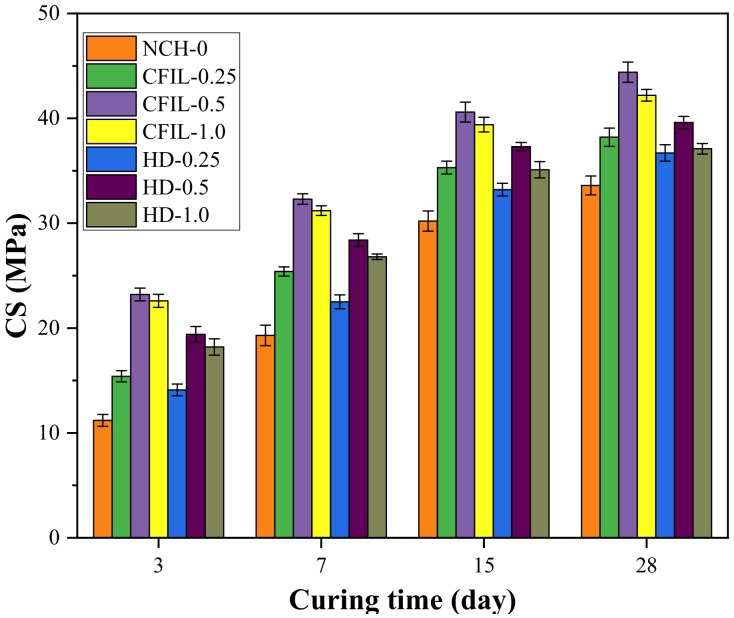
Relationship between CS and curing time.

Figure 3 shows the relationship between the CS of each experimental group and the curing time. With time, the CS of the CFIL60 and HD experimental groups increased; however, the increasing trend slowed. The change trend was the same as that of the NCH-0 experimental group. Similarly, this was mainly because the CS of the cement slurry gradually increased with the curing time. Meanwhile, the CS of the CFIL60 and HD experimental groups was significantly higher than that of the NCH-0 experimental group for the same curing time. This is because ARGF hindered the occurrence and development of cracks, reduced the porosity, and improved the CS of the slurry. Meanwhile, the CS of the CFIL60 and HD experimental groups reached a maximum for a mixing amount of 0.5%.

**Figure 4:**
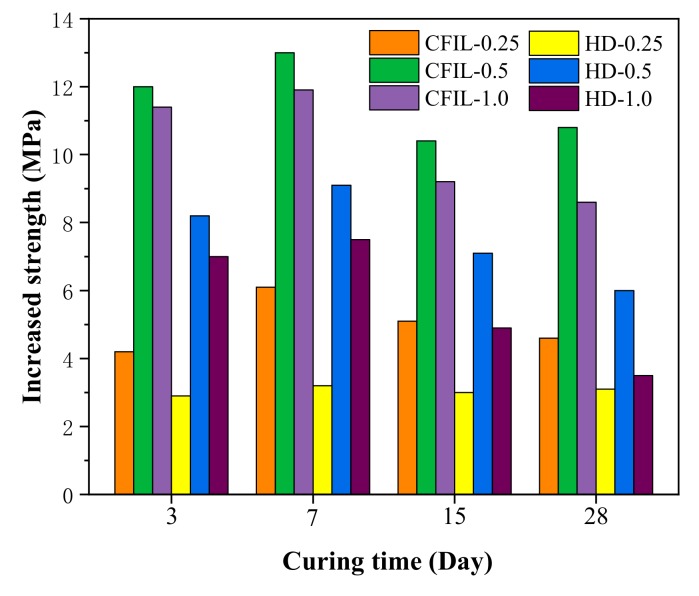
Increase in CS relative to NCH-0 for each ARGF experimental group.

Figure 4 shows the increase in CS relative to NCH-0 for each ARGF experimental group. After ARGF was added, the CS significantly improved at all curing times, and the CS increased first and then decreased with an increase in the mixing amount, with the increase in CS reaching a maximum for a mixing amount of 0.5%.

**Figure 5:**
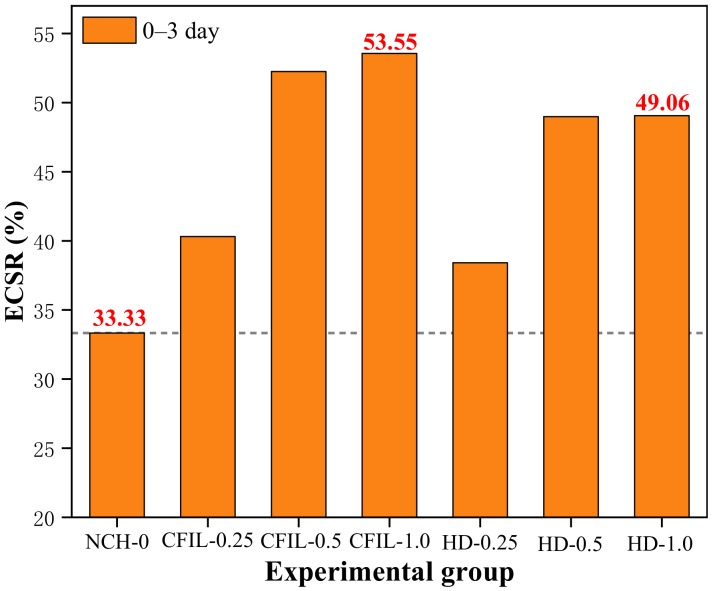
Early strength ratio of CS (ECSR) in each experimental group.

Figure 5 shows the ratio of 3 d to 28 d CS for each experimental group, which is the early strength ratio of CS (ECSR). A larger ECSR denotes that the material experiences the compressive effect earlier. ECSR has an important guiding effect on the practical engineering application of grouting slurry. As can be seen from Figure 4, adding ARGF could improve the ECSR of the slurry, as ARGF prevented the occurrence of cracks. For a mixing amount of 1.0%, the ECSR of the CFIL60 and HD experimental groups reached a maximum of 53.55% and 49.06%, respectively; meanwhile, the ECSR of NCH-0 was only 33.33%. For a mixing amount of 0.5%, ECSR was only slightly lower than when the mixing amount was 1.0%. Meanwhile, the CFIL60 group had a better ECSR than that of the HD group, indicating that the Cem-FIL60 ARGF had a better effect on ECSR.

**Figure 6:**
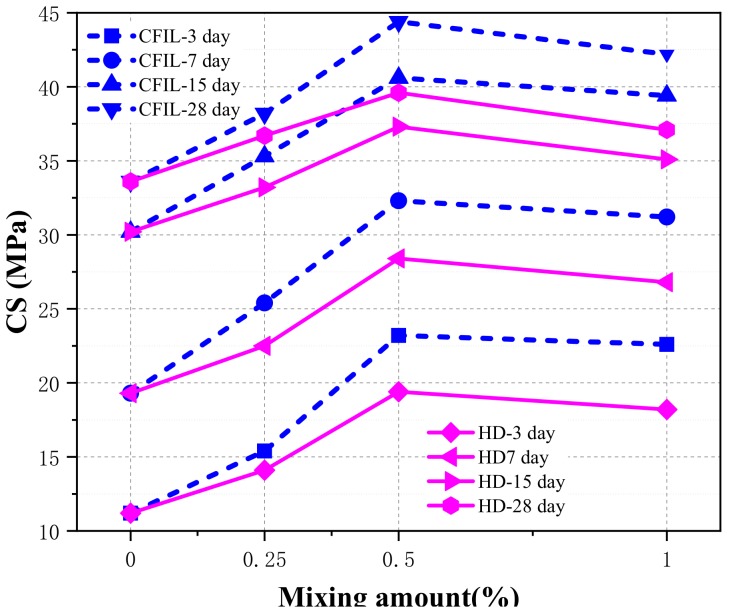
Relationship between CS and mixing amount.

Figure 6 shows the relationship between CS and the mixing amount under different curing times. With the increase in the amount, the CS of the CFIL60 and HD experimental groups increased first, then decreased, and reached the maximum when the mixing amount was 0.5%. Meanwhile, the CS of the CFIL60 experimental group was significantly better than that of the HD experimental group for the same mixing amount, indicating that the Cem-FIL60 ARGF had a better effect on the CS of the slurry than the HD material.

### 3.2. Experimental Results of TS

**Figure 7:**
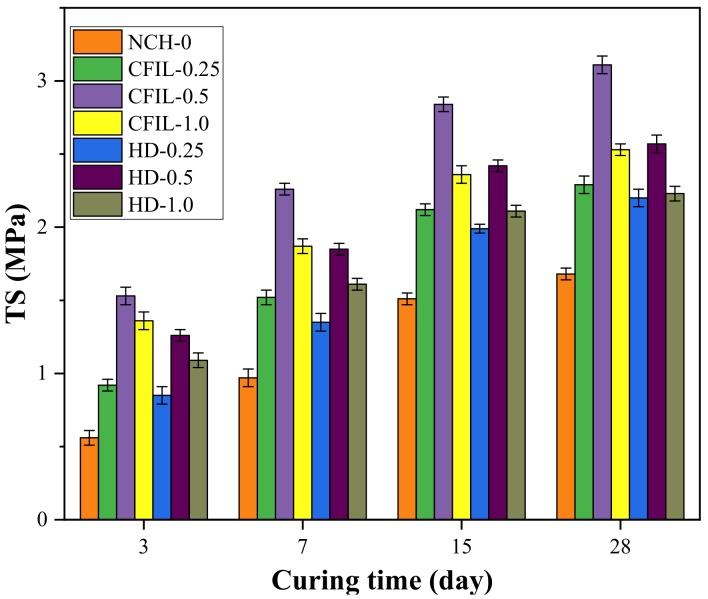
Relationship between TS and curing time.

Figure 7 shows the relationship between TS and the curing time of the experimental groups. As the curing time increased, the tensile strength of the CFIL60 and HD experimental groups also increased; however, the increasing trend slowed down, which was similar to that of the NCH-0 experimental group. The TS of CFIL60 and HD was significantly better than that of the NCH-0 for the same curing time, indicating that the addition of ARGF promoted the improvement of the TS of the slurry, and, when the mixing amount was 0.5%, the TS of the CFIL60 and HD experimental groups reached a maximum.

**Figure 8:**
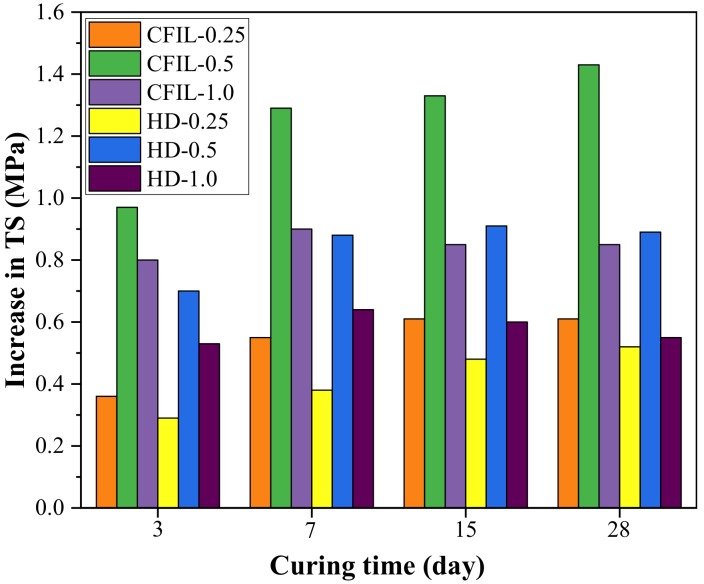
Increase in TS relative to NCH-0 for each experimental group.

Figure 8 shows the increase in TS relative to NCH-0 for each ARGF experimental group. After the addition of ARGF, the TS of CFIL60 and HD at all curing times improved significantly, and the increase in TS showed a trend of firstly increasing and then decreasing with an increase in the mixing amount. For a mixing amount of 0.5%, the increase in TS reached a maximum.

**Figure 9:**
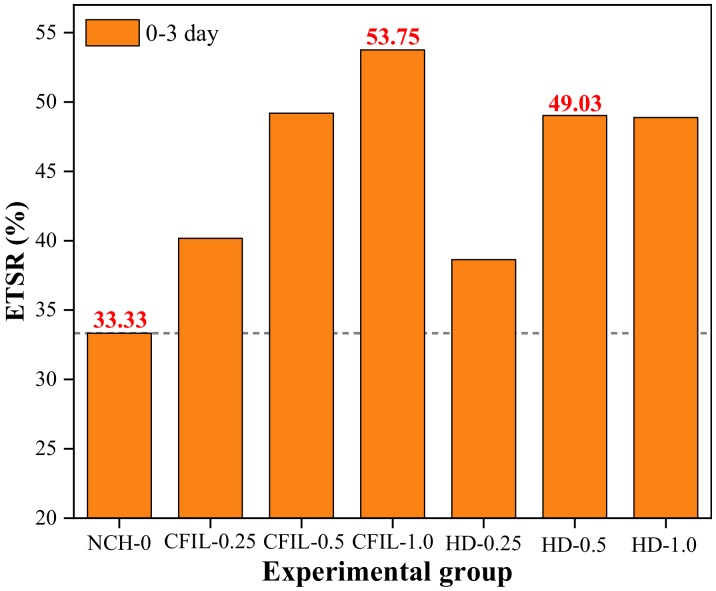
Early strength ratio of TS (ETSR) for each experimental group.

Figure 9 shows the ETSR of each experimental group. The ETSR of the slurry increased after the addition of ARGF. Moreover, for a mixing amount of 1.0% for CFIL60, the ETSR reached 53.75%. For an HD amount of 0.5%, the ETSR reached 49.05%, whereas the ETSR of NCH-0 was only 33.33%. Thus, CFIL60 had a better ETSR than that of HD for the same mixing amount, indicating that the Cem-FIL60 ARGF had a better ETSR for the slurry tensile strength than the HD material.

**Figure 10:**
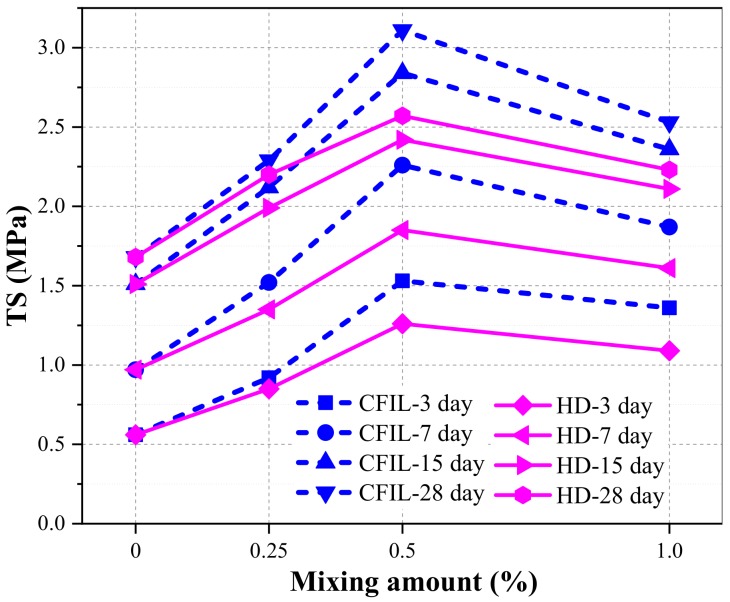
Relationship between TS and mixing amount.

Figure 10 shows the relationship between TS and the mixing amount for different curing times. With the continuous increase in the mixing amount, TS increased first and then decreased. Furthermore, the TS of CFIL60 and HD was best for a mixing amount of 0.5%. Meanwhile, the TS of CFIL60 was slightly better than that of the HD type under the same mixing amount, which showed that Cem-FIL60 ARGF had a better effect on the TS of the grout than the HD material.

### 3.3. Experimental Results of FS

**Figure 11:**
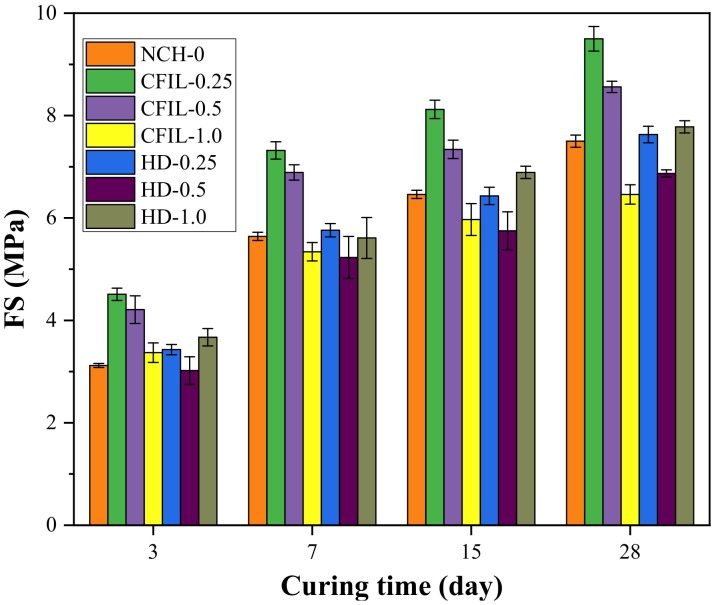
Relationship between FS and curing time.

Figure 11 shows the relationship between the FS and the curing time for each experimental group. With an increase in curing time, the FS of the CFIL60 and HD experimental groups also increased; however, the increasing trend slowed down, and the change pattern was similar to that of the NCH-0 experimental group. Moreover, the FS of CFIL60 and HD reached a maximum for mixing amounts of 0.25% and 1.0%, respectively. However, FS was lower than that of NCH-0 under certain mixing amounts.

**Figure 12:**
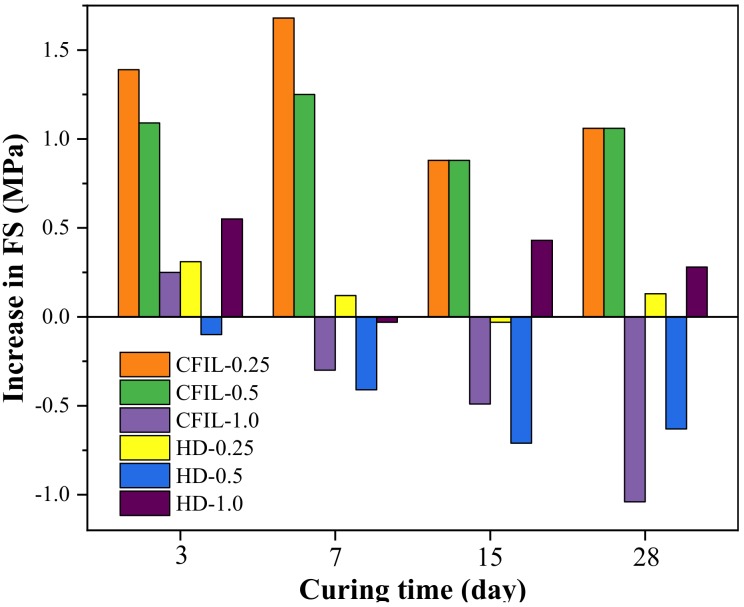
Increase in FS relative to NCH-0 for each ARGF experimental group.

Figure 12 shows the increase in FS relative to NCH-0 for each ARGF experimental group. It can be clearly seen that the FS of CFIL60 and HD was less than that of NCH-0 for mixing amounts of 1.0% and 0.5%, respectively. Therefore, the effect of the addition of ARGF on FS of the slurry was related to the mixing amount and type.

**Figure 13:**
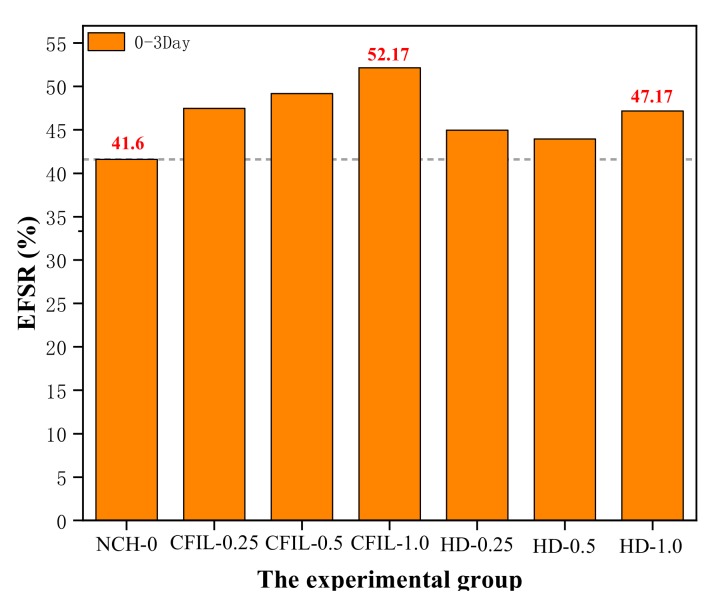
Early strength ratio of FS (EFSR) for each experimental group.

Figure 13 shows the EFSR in each experimental group. The EFSR of CFIL60 and HD experimental groups reached the highest strength ratio at 52.17% and 47.14%, respectively, when the mixing amount was 1.0%, while the EFSR of NCH-0 was 41.6%. However, although mixing with ARGF increased the EFSR of CFIL60 and HD, it may not have necessarily increased the FS. For example, when the mixing amount of the HD group was 0.25%, the EFSR was higher than that of the NCH-0 group, but the FS was lower than that of NCH-0 (comparing Figure 11 and Figure 12). Meanwhile, for the same mixing amount, the EFSR of CFIL60 was better than that of HD, indicating that the Cem-FIL60 ARGF had a better effect on the EFSR of the slurry than the HD material.

**Figure 14:**
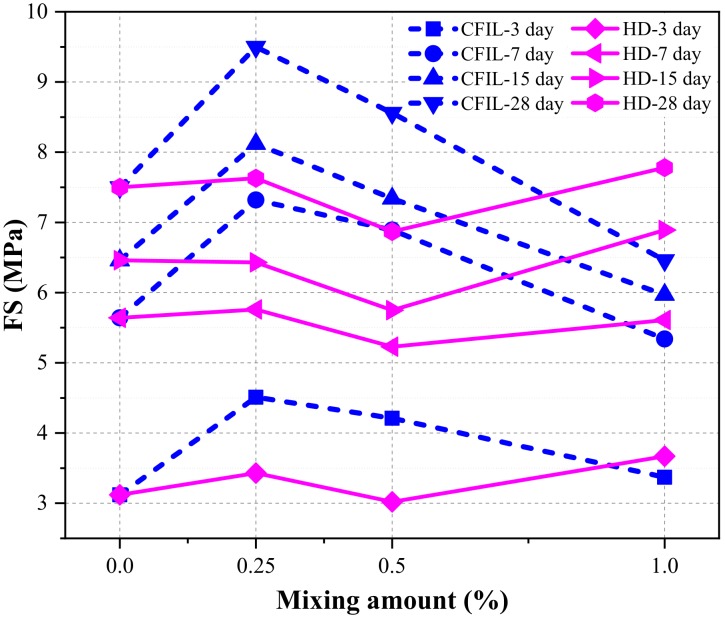
Relationship between FS and mixing amount.

Figure 14 shows the relationship between FS and the mixing amount under different curing times. With an increase in the amount of ARGF, the FS of CFIL60 first increased, then decreased, and reached a maximum when the amount was 0.25%; meanwhile, the FS of HD showed a trend of increasing first, then decreasing, and finally increasing again, reaching a maximum when the amount was 1.0%. Meanwhile, the FS of CFIL60 was better than that of HD when the mixing amount was 0.25% and 0.5%, and the FS of HD was better than that of CFIL60 when the amount was 1.0%. This shows that the effect of ARGF types on FS was directly related to the mixing amount.

### 3.4. Experimental Results of IP

The penetration height (PH) is an index used to characterize the IP. A larger PH denotes a worse IP, and, as PH decreases, the IP of ARGF improves.

**Figure 15:**
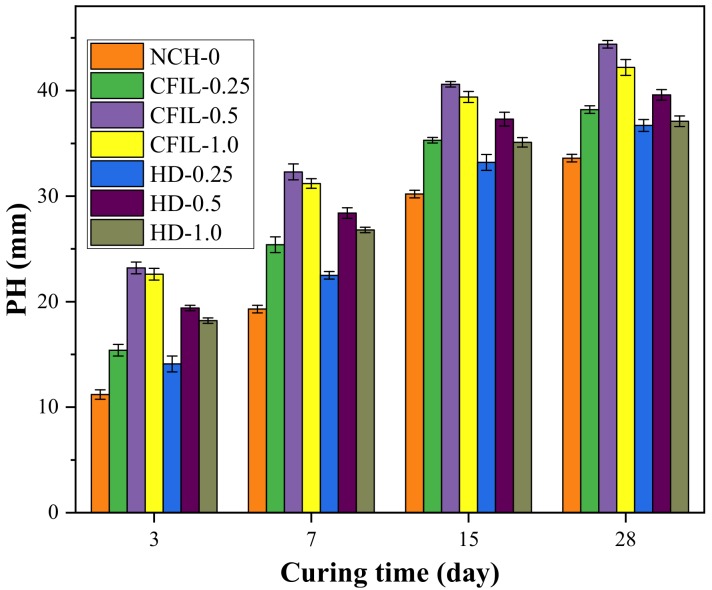
Relationship between penetration height (PH) and curing time.

Figure 15 shows the relationship between PH and curing time for each experimental group. The PH of the CFIL60 and HD testing groups decreased with curing time, while the decrease in PH slowed down, indicating that the IP also increased with curing time. The change law was similar to that of the NCH-0 experimental group. Meanwhile, CFIL60 and HD reached a minimum PH when the mixing amount was 0.5%; however, the PH was higher than that of NCH-0 for any mixing amount.

**Figure 16:**
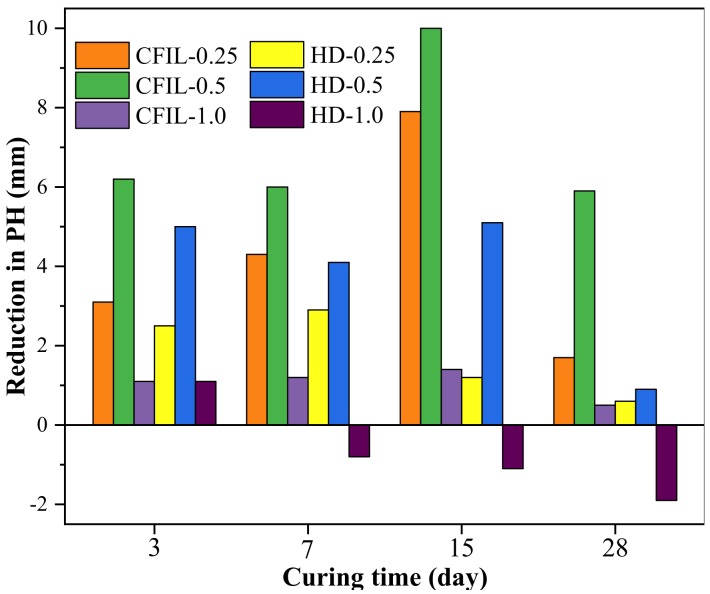
Decrease in PH relative to NCH-0 of each ARGF experimental group.

Figure 16 shows the decrease in PH relative to NCH-0 for each ARGF experimental group. With an increase in the mixing amount, the increase in the IP of CFIL60 and HD relative to NCH-0 showed a behavior of firstly increasing, then decreasing, and the gain effect was optimal when the mixing amount was 0.5%. Furthermore, the impermeability of HD ARGF at 7 d, 15 d, and 28 d was less than that of NCH-0 for a mixing amount of 1.0%. Therefore, the incorporation of ARGF may have adverse effects under certain mixing amounts and curing time conditions.

**Figure 17:**
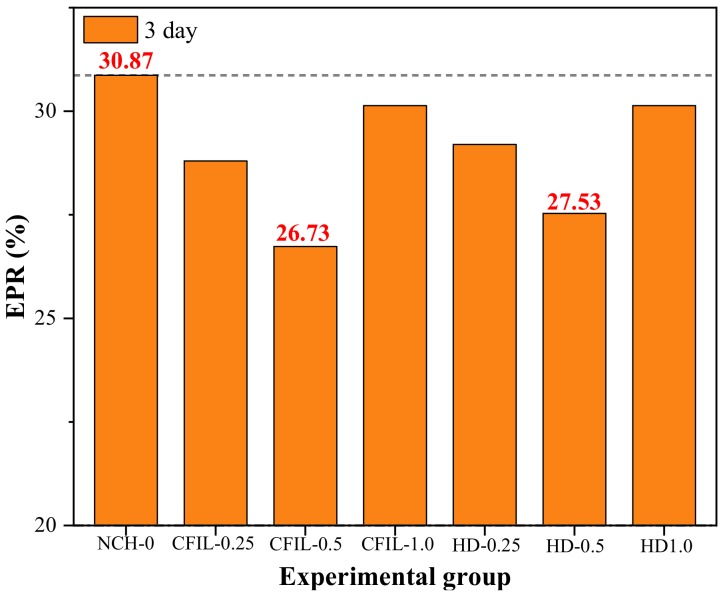
Early permeant ratio (EPR) in each experimental group.

Figure 17 shows the ratio of the PH to specimen height (specimen height was 150 mm), known as the EPR, for a curing time of 3 d. After ARGF was added, the EPR was less than that of NCH-0 for all cases, indicating that the addition of ARGF had a significant effect on improving the early IP. However, although the incorporation of ARGF reduced the EPR of CFIL60 and HD experimental groups, the result was not necessarily the improvement of the grout IP. For example, for an HD mixing amount of 1.0%, EPR was lower than that for the NCH-0 group at 7–28 d; however, the PH was higher than that of NCH-0 (Figure 15 and Figure 16). CFIL60 and HD exhibited the lowest EPR for a mixing amount of 0.5%, which indicates that the optimal IP was reached at a mixing amount of 0.5%. Meanwhile, under the same mixing amount conditions, CFIL60 exhibited a lower EPR than HD. Therefore, the early IP of Cem-FIL60 ARGF was much better than that of the HD material.

**Figure 18:**
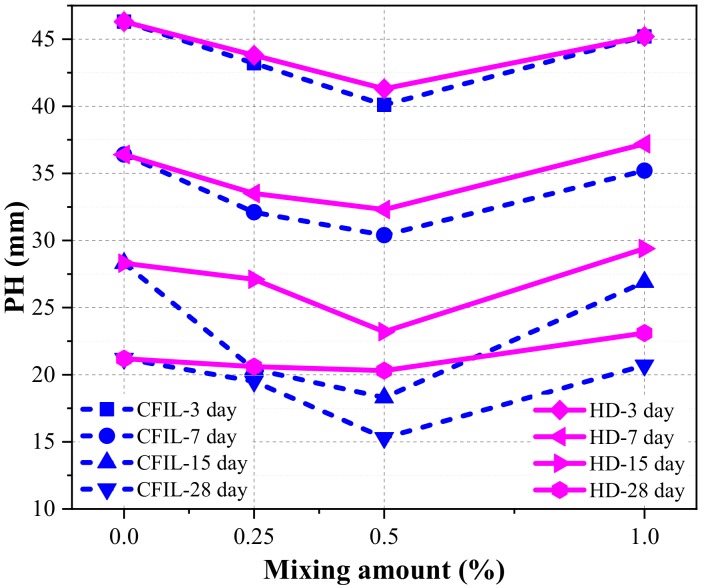
Relationship between PH and the mixing amount.

Figure 18 shows the relationship between PH and the mixing amount under different curing time conditions. With an increasing mixing amount of ARGF, the PHs of both CFIL60 and HD decreased first and then increased. Therefore, the IP firstly showed a positive correlation and then a negative correlation, and the gain effect of IP was most prominent for an ARGF mixing amount of 0.5%. Meanwhile, under the same the curing time conditions, the PH of CFIL60 was lower than that of HD. Therefore, the IP of the Cem-FIL60 ARGF is better than that of the HD material.

In summary, the analysis of the performance indicators of the slurry showed that, based on the strength analysis, the CS and TS of ARGF improved significantly, and the effect of FS and IP was directly related to its mixing amount. For example, for an HD mixing amount of 1.0%, a negative effect on FS was realized. Based on the analysis of the early strength ratios, adding ARGF increased the ECSR, ETSR, and EFSR of the grouting materials; however, an increase in EFSR did not necessarily correspond to an improvement in FS. Based on the PH ratio analysis, adding ARGF reduced the EPR and improved the IP in the early stage. Based on the analysis of the mixing amount, the CS and TS increased first and then decreased with an increase in the mixing amount. However, how FS and IP vary with mixing amount is uncertain, and some amounts were shown to have the opposite effect. Based on the material type analysis, Cem-FIL60 ARGF is superior to HD materials in terms of CS, TS, and IP, while both materials have their own advantages in terms of FS. However, in general, the effect of adding the Cem-FIL60 ARGF is better than that of the HD material.

## 4. Engineering Applications

### 4.1. Overview of Qiyueshan Tunnel Project

**Figure 19:**
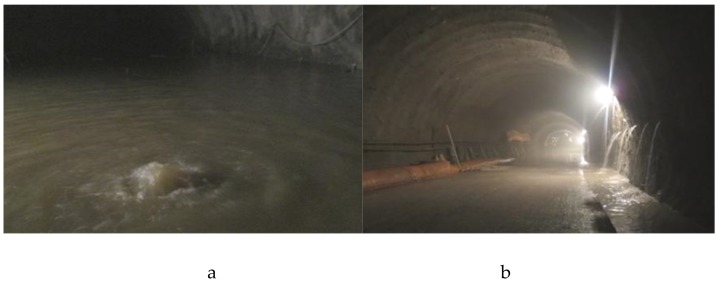
Photographs of water gushing from the tunnel scene: (**a**) normal bottom flush; (**b**) fracture-type normal water.

Qiyueshan Tunnel is a controlled project of Liwan Expressway in Lichuan City, Hubei Province. The area is mainly dominated by limestone development, with shale development in the middle. Both areas are extremely fragmented rock masses and well-developed karsts. Serious karst water gushing can be found at the excavation exposure, as shown in Figure 19.

Tunnel water gushing is the result of complex geological conditions such as karst development and geological structure fragmentation; the karst geological condition is hidden and cannot be directly revealed for processing. In this case, grouting is an effective and direct solution.

### 4.2. Grouting Scheme Design

**Figure 20:**
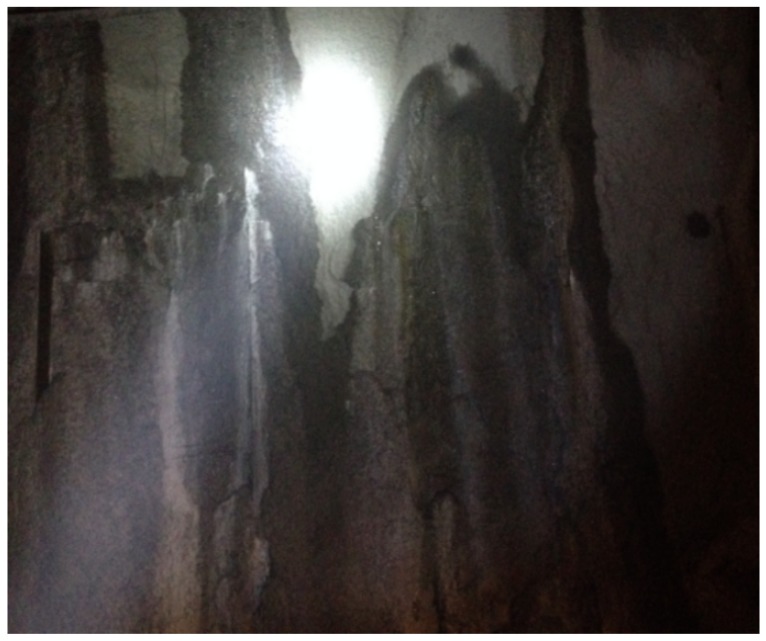
Grouting effect of pure cement slurry.

The original grouting scheme adopted pure cement grouting, in which the grout was diluted and washed out by karst water. After the grouting, large-scale water seepage (or even effluent) occurred in the surrounding rocks, and the anti-alkali phenomenon was serious, resulting in poor grouting, as shown in Figure 20. Therefore, grouting should be used not only to strengthen the surrounding rocks but also to block the water passage to prevent the occurrence of water gushing and collapse of the broken surrounding rock. Through testing, we know that the C-ARGF grouting material has good early strength and impermeability, which meets the grouting requirements of this project. According to the analysis of the experimental results, Cem-FIL60 ARGF had a significant gain effect on CS, TS, and IP when the mixing amount was 0.5%. Therefore, Cem-FIL60 ARGF was selected for this project, and its mixing amount was 0.5% with a water–cement ratio of 1:1.

**Figure 21:**
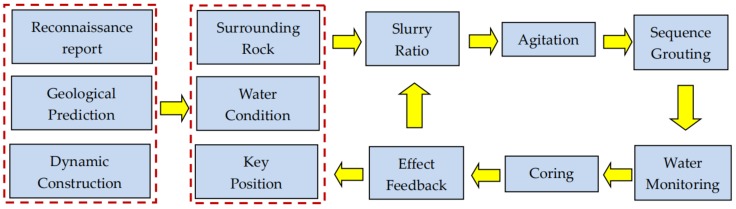
Grouting construction technology.

Owing to the complicated development of karst and fissures, the large amounts of gushing water, the richness of the karst water, and a certain dynamic pressure, the grouting pressure was selected to be 3.0–5.0 MPa, and the diffusion radius was 1.1–1.3 m. When the single-hole grouting reached the design pressure and was maintained for 5 min, or when the deformation of the surrounding rock exceeded the allowable value, the grouting was stopped. For this project, the grouting reinforcement process was carried out as shown in Figure 21.

### 4.3. Grouting Treatment Effect and Analysis

**Figure 22:**
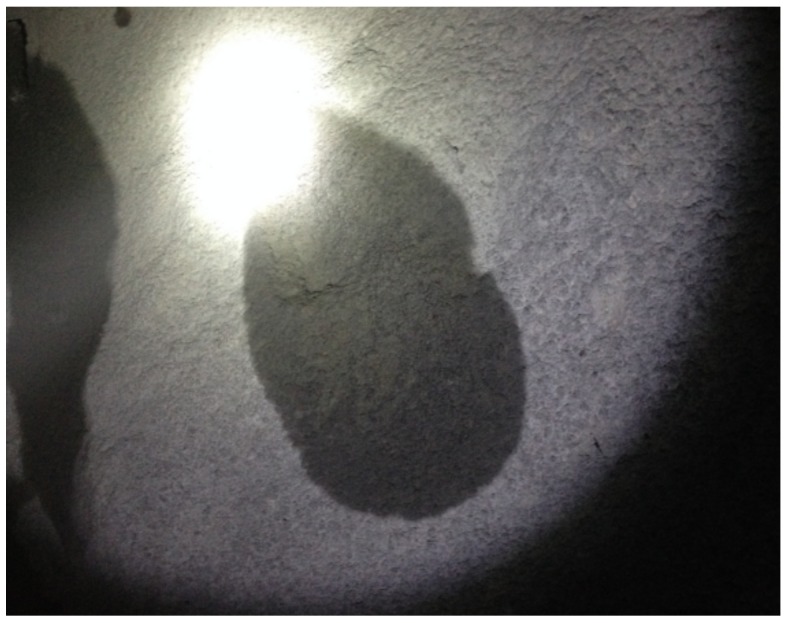
CFIL60 ARGF grouting anti-seepage effect.

After grouting, the phenomenon of infiltration occurred only in local areas without large-scale infiltration or dripping (Figure 22), and the water-stopping effect of the tunnel surrounding rock was significant.

**Figure 23:**
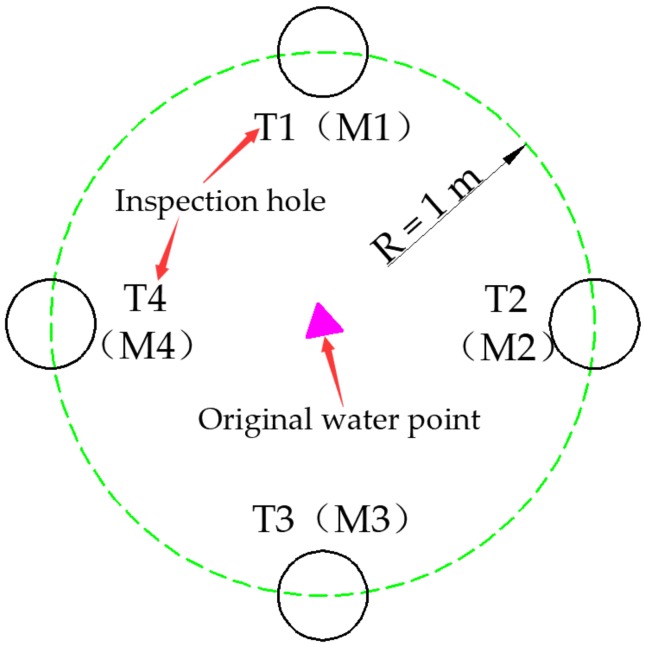
Check hole layout.

**Figure 24:**
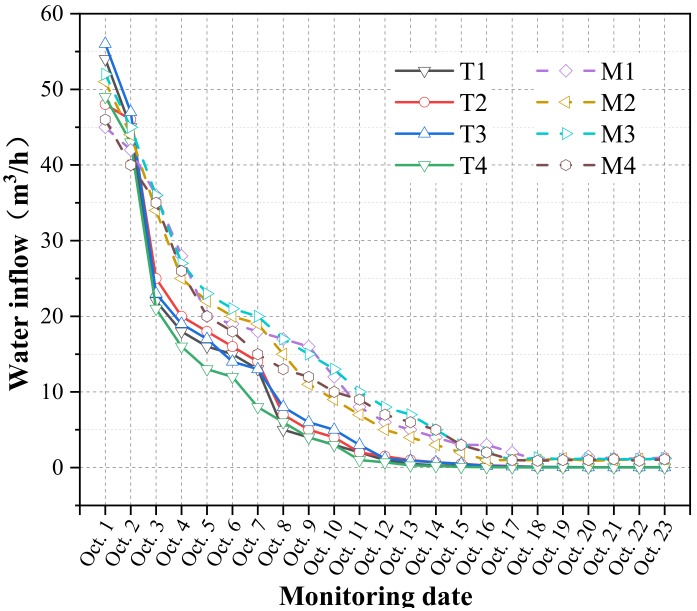
Water inflow curve of inspection hole.

In order to test the effect of grouting and water plugging and make the inspection hole representative, the inspection hole was set near the first layer of grouting circle formed around the water outlet point, and four detection holes were provided from the periphery to the water outlet point. CFIL60 ARGF grouting inspection holes were numbered T1–T4, and pure cement slurry inspection holes were numbered M1–M4. The specific layout is shown in Figure 23 below. The water inflow after grouting was monitored separately, and the frequency was set as every 2 d. The specific monitoring data are shown in Figure 24.

As can be seen from Figure 22, the water output of the T1–T4 inspection holes was effectively controlled after grouting with good effect. The water output decreased from a maximum of 56 m³/h to 0.2 m³/h, and the treatment effect was very significant. The water output of the M1–M4 grouting holes was ultimately kept under control; however, the water output decreased from a maximum of 52 m³/h to 0.9 m³/h, which was 4.5 times that of pure cement grouting, and the grouting effect was relatively poor. From the overall grouting effect, the water blocking treatment was roughly divided into three stages. The first stage was the rapid decline stage. Within 48 h of the grouting, the cement-based glass fiber slurry quickly played an important role in blocking the water. The water inrush was clearly restrained. The water-stopping speed of the Cem-FIL60 ARGF grouting was faster than that of the pure cement slurry. This had a direct relationship with the early strength and early impermeability of the grouting material after the addition of ARGF. In the second stage, the water output decreased steadily. Owing to the blocking of the water outlet point, the slurry further solidified, the water inflow further decreased, and the trend of the change in the water output gradually slowed. At this stage, the Cem-FIL60 ARGF grout exhibited a better water-stopping effect. The third stage demonstrated stability. After the slurry reached the final setting strength, the full effect was exerted. The Cem-FIL60 C-ARGF grouting material reached a stable state earlier than that of the pure cement slurry grouting, and the water output was reduced, demonstrating the significant blocking effect.

## 5. Conclusions

The use of ARGF as an additive material of grouting slurry was examined. Through the experiment, the influence of the types and mixing amounts of ARGF on the CS, TS, FS, and IP of the slurry was studied. Meanwhile, the influence of the types and mixing amounts of ARGF on the ECSR, ETSR, EFSR, and EPR of the slurry was analyzed. The mixing amounts of the optimal volume and types of ARGF were determined, providing a basis for the engineering application of this grouting material.

(1) The addition of ARGF was found to improve the CS and TS of the grouting material, and the CS and TS of Cem-FIL60 and HD ARGF reached a maximum when the mixing amount was 0.5%.

(2) Different mixing amounts of ARGF were shown to have different effects on the FS, even resulting in a counteracting effect at certain amounts. For example, for mixing amounts of Cem-FIL60 and HD of 1.0% and 0.5%, respectively, the FS was lower than that of the pure cement slurry for the same curing time. For mixing amounts of Cem-FIL60 and HD of 0.25% and 1.0%, respectively, the FS reached a maximum, along with the IP. For a mixing amount of HD of 1.0, the PH was higher than that of pure cement slurry at 7–28 d; however, this phenomenon only occurred for an HD mixing amount of 1.0%.

(3) Mixed ARGF resulted in increased ECSR, ETSR, and EFSR. Meanwhile, the CS and TS increased along with the rise of ECSR and ETSR. However, as for FS, it was lower than that of the pure cement slurry, although the EFSR increased under certain mixing amounts. Mixed ARGF could reduce the EPR of the slurry; however, the reduction in EPR did not represent an improvement in the IP. Under some mixing amounts, the EPR decreased, while the IP was weaker than that of pure cement slurry.

(4) In comparing the superiority between the Cem-FIL60 and HD ARGFs, for mixing amounts of 0.25% and 0.5%, Cem-FIL60 was shown to be better than HD in CS, TS, and IP. However, for a mixing amount of 1.0%, HD was shown to be better than Cem-FIL60 in FS. Therefore, Cem-FIL60 ARGF was revealed to be generally superior to HD. Thus, CFIL60 ARGF can be the initial choice for mixing material in grouting engineering applications. 

Some problems were found in the application of C-ARGF grouting material, and the most common problem was pipe plugging. After field analysis, it was found that the problem is mainly related to the length and mixing amount of ARGF. In order to ensure the application effect of the new grouting material, the best combination of length and dosage of ARGF should be implemented to solve this problem in the future.

## Figures and Tables

**Table 1:** Type and parameters of ARGF.

Species	The Length (mm)	Equivalent Diameter (μm)	Fracture Strength (MPa)	Elongation at Break (%)	Modulus (GPa)	Melting Point (°C)
Cem-FIL 60	12	14	1700	3.6	72	1580
HD	12	14	1700	3.6	72	1580

**Table 2:** Main test equipment. CS—compression strength; TS—tensile strength; FS—flexural strength; IP—impervious performance.

Name	Model	Precision Value for	Use	Appended Figures
Electro-hydraulic pressure testing machine	TYA-2000	±1%	CS test	Figure 2b
Flexural and Compressive tester	SYE-300	±1%	TS test	Figure 2c,d
			FS test	
Program-controlled automatic pressure regulating impermeable instrument	HP-4.0	0.3% FS	IP test	Figure 2e

**Table 3:** Experimental group design table.

Project	Curing Time	ARGF Mixing Amount (volume %)						
		NCH-0	CFIL-0.25	CFIL-0.5	CFIL-1.0	HD-0.25	HD-0.5	HD-1.0
		0	0.25	0.5	1.0	0.25	0.5	1.0
CS	3 d	A B C	A B C	A B C	A B C	A B C	A B C	A B C
	7 d	A B C	A B C	A B C	A B C	A B C	A B C	A B C
	15 d	A B C	A B C	A B C	A B C	A B C	A B C	A B C
	28 d	A B C	A B C	A B C	A B C	A B C	A B C	A B C
TS	3 d	A B C	A B C	A B C	A B C	A B C	A B C	A B C
	7 d	A B C	A B C	A B C	A B C	A B C	A B C	A B C
	15 d	A B C	A B C	A B C	A B C	A B C	A B C	A B C
	28 d	A B C	A B C	A B C	A B C	A B C	A B C	A B C
FS	3 d	A B C	A B C	A B C	A B C	A B C	A B C	A B C
	7 d	A B C	A B C	A B C	A B C	A B C	A B C	A B C
	15 d	A B C	A B C	A B C	A B C	A B C	A B C	A B C
	28 d	A B C	A B C	A B C	A B C	A B C	A B C	A B C
IP	3 d	A B C D E F	A B C D E F	A B C D E F	A B C D E F	A B C D E F	A B C D E F	A B C D E F
	7 d	A B C D E F	A B C D E F	A B C D E F	A B C D E F	A B C D E F	A B C D E F	A B C D E F
	15 d	A B C D E F	A B C D E F	A B C D E F	A B C D E F	A B C D E F	A B C D E F	A B C D E F
	28 d	A B C D E F	A B C D E F	A B C D E F	A B C D E F	A B C D E F	A B C D E F	A B C D E F

*Note:* In terms of the CS, TS, and FS tests, the experimental group contained three specimens: A, B, and C. However, each experimental group contained six specimens in the impermeability test; the specimens were A, B, C, D, E, and F.

## Nomenclature

**Cem-FIL60**	Cem-FIL®60 is a high cluster alkali-resistant glass staple fiber.
**HD**	Anti-Crak®HD (high dispersion) is an engineered AR glass chopped strand designed for mixing in concrete and all hydraulic mortars.
**ARGF**	Alkali-resistant glass fiber
**C-ARGF**	Cement alkali-resistant glass fiber
**CS**	Compressive strength
**TS**	Tensile strength
**FS**	Flexural strength
**IP**	Impervious performance
**PH**	Penetration height.
**ECSR**	Ratio of 3 d to 28 d CS for each experimental group, the early strength ratio of CS, which is abbreviated as ECSR.
**ETSR**	Ratio of 3 d to 28 d TS for each experimental group, the early strength ratio of TS, which is abbreviated as ETSR.
**EFSR**	Ratio of 3 d to 28 d FS for each experimental group, the early strength ratio of FS, which is abbreviated as EFSR.
**EPR**	Ratio of penetration height to specimen height (specimen height is 150 mm) at the curing time of 3 d. This ratio is called the early permeant ratio, which is abbreviated as EPR.
